# Purinergic Activation of Store-Operated Calcium Entry (SOCE) Regulates Cell Migration in Metastatic Ovarian Cancer Cells

**DOI:** 10.3390/ph16070944

**Published:** 2023-06-29

**Authors:** Esperanza Mata-Martínez, Adriana Gonzalez-Gallardo, Mauricio Díaz-Muñoz, Francisco G. Vázquez-Cuevas

**Affiliations:** 1 Departamento de Neurobiología Celular y Molecular, Universidad Nacional Autónoma de México, Boulevard Juriquilla#3001, Juriquilla 76230, Querétaro, Mexico; espemmtz@gmail.com; 2 Unidad de Proteogenómica, Instituto de Neurobiología, Universidad Nacional Autónoma de México, Boulevard Juriquilla#3001, Juriquilla 76230, Querétaro, Mexico; gallardog@unam.mx

**Keywords:** purinergic signaling, store-operated calcium entry, P2Y2 receptor, ovarian carcinoma, SKOV-3, cell migration

## Abstract

Store-operated calcium entry (SOCE) is an important process in calcium signaling. Its role in physiological and pathological events is well recognized. However, in cancerous systems, the importance of SOCE in relation to the degree of cancer aggressiveness, as well as its regulation by ligands such as purinergic molecules, are not well documented. This study aimed to characterize a differential effect of the P2Y2 receptor (promoted by UTP of 10 µM and inhibited by ARC118925XX of 1 µM) on intracellular calcium response between metastatic (SKOV-3) and non-metastatic (CAOV-3) ovarian cell lines in conditions of normal (1.5 mM) and zero extracellular calcium concentration. The sustained calcium influx observed exclusively in SKOV-3 cells was associated with the presence of SOCE (promoted by thapsigargin (74.81 ± 0.94 ΔF) and sensitive to 2-APB (20.60 ± 0.85 ΔF)), whereas its absence in CAOV-3 cells (26.2 ± 6.1 ΔF) was correlated with a low expression of ORAI1. The relevance of SOCE in metastatic SKOV-3 cells was further corroborated when 2-APB significantly inhibited (40.4 ± 2.8% of covered area) UTP-induced cell migration (54.6 ± 3.7% of covered area). In conclusion, our data suggest that SOCE activation elicited by the P2Y2 receptor is involved in the aggressiveness of ovarian cancer cells.

## 1. Introduction

Ovarian carcinoma (OvCa) is the most lethal gynecological disease [1]; this lethality is caused in part by its high metastatic potential, supported by a particular mechanism named transcoelomic dissemination that involves phenotypic changes in cancer cells orchestrated by a variety of factors within the tumor microenvironment (TME) [2]. Therefore, understanding the TME-dependent mechanisms regulating cell migration in metastatic OvCa cells may contribute to the definition of oncogenic processes, and eventually to the discovery of new therapeutic targets.

The purinergic system has been recognized as an important element of TME; for example, actions of extracellular ATP and its metabolites contribute to tumor progression [3] since cancer cells exhibit adaptations in diverse elements that confer efficiency to purinergic signaling. Metabolic adaptations, such as the Warburg effect, make glycolytic ATP synthesis efficient [4]; in cancerous cells, the overproduction of intracellular ATP favors nucleotide release to the extracellular space. It is well established that extracellular concentration of ATP in tumor stroma is enhanced in comparison with healthy tissues (mM vs. nM levels) [5]; accordingly, tumor tissues express diverse functional purinergic receptors [6]. Consequently, autocrine–paracrine activation of purinergic receptors may regulate events in tumor cells such as cell trans-differentiation, proliferation and migration [3,6].

Extracellular ATP exerts its actions through the P2 receptor family, which is composed of two subfamilies: P2X, which are ligand-activated ion channels, and P2Y, which belong to class A of the G-protein-coupled receptor (GPCR) superfamily. A subgroup of P2Y receptors (P2Y1, P2Y2, P2Y4 and P2Y11) are coupled to Gq proteins, whose canonical transduction pathway consists of the activation of phospholipase C, synthesis of IP_3_ and diacylglycerol, and an increment in intracellular Ca^2+^ concentration ([Ca^2+^]_i_) through IP_3_ receptors located in internal calcium stores [7]. From this receptor subgroup, P2Y2 has been deeply characterized in an oncogenic context. 

P2Y2 receptor activation induces cell proliferation in carcinoma cells from various tissues such as lung, ovary, breast, prostate and cervix [8,9,10,11,12,13], and also regulates cell migration and epithelial-to-mesenchymal transition induction in breast, prostate, ovary and gastric carcinoma cells [12,14,15,16,17]. Despite the important role of P2Y2 in carcinoma, the signal transduction pathways regulated by this receptor remain partially described, particularly the mechanisms mediated by intracellular Ca^2+^.

In addition to the canonical activation of Ca^2+^ release via IP_3_ production, the P2Y2 receptor is able to activate, in adipocytes, the store-operated calcium entry (SOCE) [18], which is the most conspicuous pathway of Ca^2+^ influx in non-excitable cells. SOCE occurs when Stim1, a protein resident in the membrane of the endoplasmic reticulum, senses a reduction in Ca^2+^ levels within the lumen of this organelle and changes its conformation to interact with Orai1, a Ca^2+^ channel located in the plasma membrane that initiates an associated Ca^2+^ influx [19].

Previous reports have demonstrated that SOCE is critical for cell migration and metastasis induction in breast cancer, where the knock-out of Stim1 or Orai1 significantly reduces migration of MDA-MB-231 cells by altering the dynamics of focal adhesion turnover that occurs during this event [20]. In this context, it is a priority to understand the molecular mechanisms that underlie oncogenic processes to propose potential therapeutic anticancer strategies [21,22]. In the present work, we examine the occurrence of SOCE elicited by P2Y2 receptor activation in OvCa-derived cell lines and demonstrate that SOCE is present in metastatic (SKOV-3) cells, but not in non-metastatic (CAOV-3) cells, which is partly coincident with the differential expression of ORAI1. Furthermore, we show that SOCE participates in P2Y2-dependent cell migration in SKOV-3 cells. 

## 2. Results

### 2.1. UTP Induces Intracellular Ca^2+^ Mobilization Dependent on Extracellular Influx and Ca^2+^ Release from Internal Deposits in Metastatic Ovarian Carcinoma Cells

As mentioned above, P2Y2/P2Y4 receptors regulate important cellular processes in cancer cells; however, the signaling mechanisms are not yet fully understood. To analyze the effects of P2Y2/P2Y4 receptor activation on intracellular Ca^2+^ concentration [Ca^2+^]_i_ dynamics in metastatic OvCa cells, SKOV-3 cells were loaded with Fluo4-AM and stimulated with UTP, and intracellular Ca^2+^ was recorded in single-cell experiments. The expression of P2Y2 receptors in the SKOV-3 cell line was previously demonstrated by our group [16]; to confirm this expression, RT-PCR with specific oligonucleotides was performed, and an amplicon of 189 base pairs was obtained, sequenced and analyzed by BLAST. The sequence was identified with the NM_176072.3 entry that corresponds to the cDNA of the human P2Y2 receptor (Figure S1A,B). Furthermore, we compared the expression level of *P2RY2* between CAOV-3 and SKOV-3 by RT-qPCR (Figure S1); SKOV-3 cells showed an increased expression of *P2R2Y* transcript (3.2 ± 0.09 fold of CAOV-3) (Figure S1C). 

**Figure 1:**
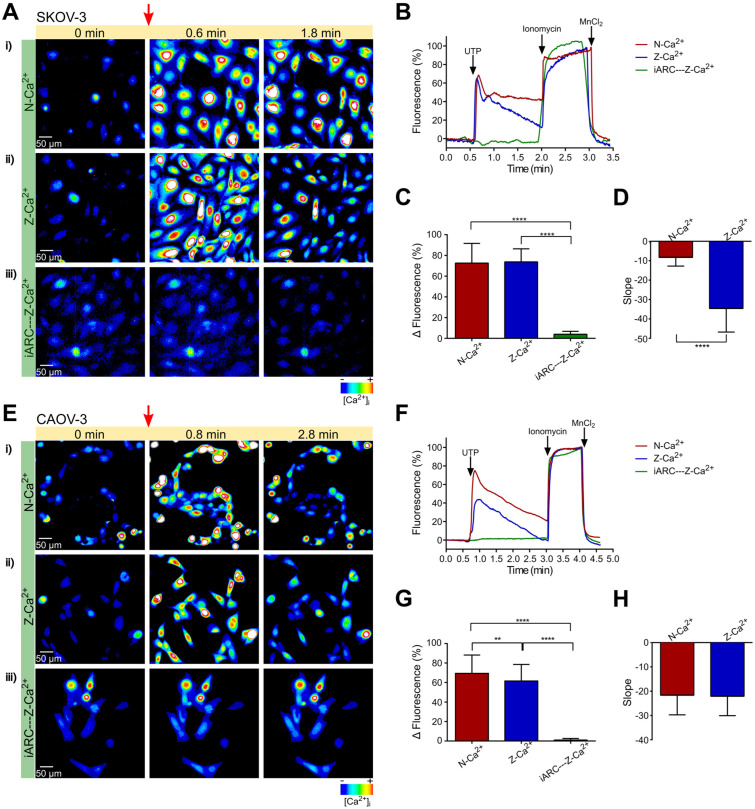
UTP elicits Ca^2+^ mobilization in metastatic ovarian carcinoma SKOV-3 cells and CAOV-3 cells. Sequence of fluorescent images from SKOV-3 cells (**A**) or CAOV-3 cells (**E**) loaded with Fluo-4 AM 2 µM showing changes in [Ca^2+^]_i_ before and after the addition of UTP of 10 µM (red arrow) in N-Ca^2+^ or Z-Ca^2+^ extracellular solutions, in presence or absence of the P2Y2 receptor antagonist ARC118925XX 1 µM (iARC); pseudocolor from black to red represents low to high [Ca^2+^]_i_, respectively; time frames are indicated in minutes. Representative Ca^2+^ fluorescence (Fluo-4) traces from SKOV-3 cells (**B**) and CAOV-3 cells (**F**) obtained in N-Ca^2+^, Z-Ca^2+^ and Z-Ca^2+^ plus ARC. Cells were stimulated with 10 μM UTP in N-Ca^2+^ or Z-Ca^2+^ extracellular solutions, in presence or absence of the P2Y2 receptor antagonist ARC118925XX 1 µM (iARC); at the end of the protocol, ionomycin (10 µM) and MnCl_2_ (5 mM) were sequentially applied to determine the maximum and minimum levels of intracellular Ca^2+^, respectively. When ionomycin was added in Z-Ca^2+^ condition, at the same time, the extracellular Ca^2+^ was restored to levels of N-Ca^2+^. (**C**,**G**) Mean ± S.D. of the maximal amplitude of the fast Ca^2+^ transient elicited by UTP and (**D**,**H**) Mean ± S.D. of the slope displayed by the sustained component of the Ca^2+^ response induced by UTP in N-Ca^2+^, Z-Ca^2+^ and Z-Ca^2+^ plus ARC observed in SKOV-3 cells and CAOV-3 cells, respectively. At least 100 cells were analyzed per experiment, n = 3. **** *p* < 0.0001, ** *p* < 0.01. Student’s *t*-test (**D**,**H**); Kruskal-Wallis and Dun´s tests as *post hoc* (**C**,**G**).

Stimulation of SKOV-3 cells with a 10 μM UTP, a concentration greater than the EC_50_ for P2Y2 but not saturating [23], in 1.5 mM of extracellular Ca^2+^ (N-Ca^2+^) induced a biphasic increment in [Ca^2+^]_i_ that consisted of a fast transient (72.6 ± 1.4 ΔF) that decreased by nearly 80% in 0.5 min, and then displayed a sustained component that did not return to basal level after 1.5 min (Figure 1A(i),B). The same stimulus in zero extracellular Ca^2+^ (Z-Ca^2+^) induced a transient with a similar amplitude (73.8 ± 0.9 ΔF) (Figure 1C), but the sustained component changed to a gradual return to basal level after 1.5 min (Figure 1A(ii) vs. Figure 1B). The comparison of the slope between N-Ca^2+^ and Z-Ca^2+^ of the sustained component of the responses is shown in Figure 1D. These results suggest that the first transient is produced by activation of the PLC-IP_3_ system, and the sustained component depends on Ca^2+^ influx.

To determine whether the UTP-dependent Ca^2+^ mobilization is mediated by P2Y2 or P2Y4 receptors, we incubated SKOV-3 cells with 1 μM of ARC118925XX before stimulus with UTP; the antagonist abolished the response elicited by UTP (73.8 ± 0.9 vs. 4.0 ± 0.2 ΔF) (Figure 1C), suggesting that this response is promoted mainly by P2Y2 receptor activation.

### 2.2. The Intracellular Ca^2+^ Mobilization Elicited by UTP in Non-Metastatic CAOV-3 Cells Is Similar in Presence or Absence of Extracellular Ca^2+^

To evaluate the Ca^2+^ dynamics elicited by P2Y2/P2Y4 activation in non-metastatic OvCa cells, CAOV-3, a cell line isolated from a non-metastatic ovarian primary tumor [24], was stimulated with a 10 μM UTP in N-Ca^2+^. The agonist elicited a fast transient (69.4 ± 2.1 ΔF) that gradually returned to basal level (Figure 1E(i),F). When the stimulus was made in Z-Ca^2+^, a response with similar kinetics but minor amplitude was observed (61.5 ± 1.8 ΔF) (Figure 1E–G). Slopes of both N-Ca^2+^ and Z-Ca^2+^ responses were similar (Figure 1H), indicating only a marginal contribution of Ca^2+^ influx. The change in [Ca^2+^]_i_ observed in Z-Ca^2+^ was abolished by preincubation with ARC118925XX (Figure 1G), suggesting that, in a similar way to metastatic OvCa cells, the response also depends on P2Y2 receptor activity.

### 2.3. Store-Operated Calcium Entry (SOCE) Is Mainly Present in Metastatic (SKOV-3) Cells

**Figure 2:**
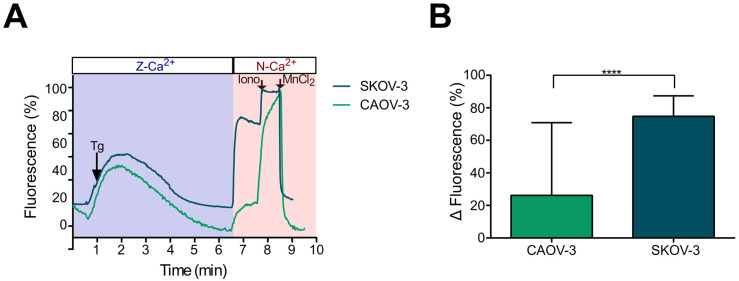
Induction of SOCE by thapsigargin in metastatic SKOV-3 and non-metastatic CAOV-3 cells. SOCE induction was monitored by Ca^2+^ image recording. To induce SOCE, cultures of SKOV-3 or CAOV-3 cells loaded with Fluo-4 AM were treated with 1 µM of thapsigargin in Z-Ca^2+^ extracellular solution. At minute 6 of recording, extracellular Ca^2+^ was restored by applying N-Ca^2+^ extracellular solution to identify the Ca^2+^ influx. At the end of the recording, ionomycin (10 μM) and MnCl_2_ (5 mM) were sequentially applied to determine the maximum and minimum levels of intracellular Ca^2+^, respectively. (**A**) Representative traces of SOCE induction by thapsigargin in SKOV-3 and CAOV-3 cells. The arrows indicate the time of drug application. (**B**) Mean ± S.D. of the increase in [Ca^2+^]_i_ after extracellular Ca^2+^ restoring in SKOV-3 and CAOV-3 cells. At least 100 cells were analyzed per experiment, n = 3. **** *p* < 0.0001, Student’s *t*-test.

Considering the differences in intracellular Ca^2+^ dynamics produced by UTP in N-Ca^2+^ and Z-Ca^2+^ observed only in SKOV-3 cells, we investigated whether the sustained component, which is probably dependent on Ca^2+^ influx, was prompted by SOCE current activation and confirmed that this current is only present in metastatic cells. Hence, the SKOV-3 and CAOV-3 cultures were recorded under a typical SOCE induction protocol [25]. Briefly, in Z-Ca^2+^, SOCE was induced by the application of a 1 μM thapsigargin. After 6 min, N-Ca^2+^ was restored with the aim of revealing the Ca^2+^ influx produced by store depletion. Finally, maximum and minimum levels of [Ca^2+^]_i_ were estimated by the sequential addition of ionomycin (10 μM) and MnCl_2_ (5 mM), respectively. In both SKOV-3 and CAOV-3 cells, thapsigargin in Z-Ca^2+^ induced a transient increment in [Ca^2+^]_i_ that returned to basal level after around 6 min (Figure 2A); however, the response to N-Ca^2+^ restoring was different between the two cell lines. In SKOV-3, Z-Ca^2+^ indicated an important increase in [Ca^2+^]_i_ (74.8 ± 0.9), while in CAOV-3, only a modest increase was observed (26.2 ± 6.1) (Figure 2A,B). 

These results suggested that thapsigargin-induced Ca^2+^ influx mediated by SOCE is mainly present in metastatic OvCa cells.

### 2.4. Molecular Components of SOCE Are Differentially Expressed in Healthy and Cancerous Cells

From the previous results, it is clear that the characteristics of Ca^2+^ dynamics elicited by P2Y2 receptor activation by UTP are different in metastatic and non-metastatic OvCa cells. In particular, the differences due to Ca^2+^ influx could be related to the expression of elements required for SOCE activation. To explore this topic, we analyzed transcripts from genes coding for molecular components of SOCE using reverse transcription and end-point polymerase chain reaction (RT-PCR) in total RNA from human ovarian surface epithelium cells (hOSE), non-metastatic CAOV-3 and metastatic SKOV-3 OvCa cells as described in the Methods section. Amplicon identities were corroborated by sequencing and analysis in the BLAST platform (NIH-USA).

**Figure 3:**
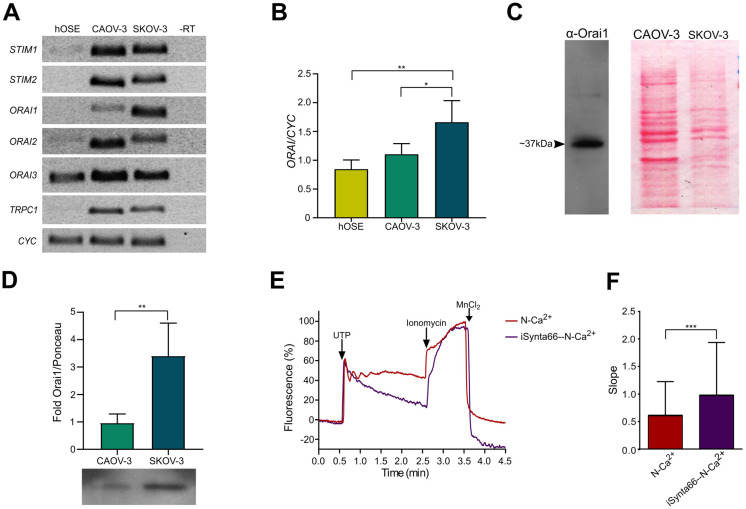
Expression of transcript from genes coding for proteins mediating SOCE. (**A**) Transcripts of *STIM1*, *STIM2*, *ORAI1*, *ORAI2*, *ORAI3* and TRPC1 were analyzed by reverse transcription and end-point polymerase chain reaction (PCR) in mRNAs from human ovarian surface epithelium cells (hOSE), SKOV-3 and CAOV-3 cells. Amplicons were visualized in a 1.2% agarose gel and their identity was confirmed by sequencing and BLAST analysis. (**B**) *ORAI1* transcript was analyzed by quantitative PCR in cDNAs from hOSE, CAOV-3 and SKOV-3 cells. (**C**) By immunoblot, ORAI1 was recognized as a single band of around 37 kDa in SKOV3 cell homogenates (left), Ponceau red stain was made for CAOV-3 and SKOV-3 homogenates (right). (**D**) Relative abundance of ORAI1 in CAOV-3 and SKOV-3 cells determined by immunoblot, abundance was corrected against Ponceau Red stain. (**E**) Representative Ca^2+^ fluorescence (Fluo-4) traces from SKOV-3 cells obtained in N-Ca^2.^. Cells were stimulated with a 10 μM UTP in N-Ca^2+^ extracellular solutions, in presence or absence of the Orai1 inhibitor Synta66 500 nM; at the end of the protocol, ionomycin (10 µM) and MnCl_2_ (5 mM) were sequentially applied to determine the maximum and minimum levels of intracellular Ca^2+^, respectively. The arrows indicate the time of drug application. (**F**) Mean ± S.D. of the slope displayed by the sustained component of the Ca^2+^ response induced by UTP in N-Ca^2+^ in presence and absence of Synta66 500 nM observed in SKOV-3 cells. At least 100 cells were analyzed per experiment, n = 3. * *p* < 0.05, ** *p* < 0.01, *** *p* < 0.001. Student’s *t*-test (**D**,**F**) and ANOVA (**B**); three independent determinations.

The first observation was that all the evaluated transcripts named, *STIM1*, *STIM2*, *ORAI1*, *ORAI2*, *ORAI3* and *TRPC1* with the exception of housekeeping genes, showed very low levels of mRNA in hOSE, whereas in CAOV-3 and SKOV-3 cells, all the amplicons were well represented (Figure 3A). Interestingly, the main difference between CAOV-3 and SKOV-3 cells was the abundance in the *ORAI1* transcript observed in SKOV-3 cells. Then, we considered the possibility that differences observed in Ca^2+^ mobilization elicited by UTP in metastatic and non-metastatic cell lines could be explained by the reduction in SOCE current activation due to the low expression in *ORAI1* in CAOV-3 cells (Figure 1). *ORAI1* transcript level was compared by quantitative PCR in RNA from hOSE, CAOV-3 and SKOV-3 cells; the results showed differential expression with the following order of abundance: hOSE < CAOV-3 < SKOV-3 (Figure 3B). To support the last observation, the relative abundance of ORAI1 in non-metastatic and metastatic cell lines was evaluated by immunoblot as described in the methods. The employed antibody detects a unique band of ~37 kDa in extracts of SKOV-3 cells (Figure 3C left). Relative expression was corrected against the Ponceau red stain signal (Figure 3C right), and the abundance of ORAI1 was greater in SKOV-3 cells than in CAOV-3 cells (3.4 ± 0.6-fold of CAOV-3) (Figure 3D), confirming our previous observations. These results suggest that expression of ORAI1 can contribute with the differential UTP-dependent induction of SOCE current in non-metastatic and metastatic cells.

To examine the role of ORAI1 in the generation of UTP-induced SOCE, we tested the effect of Synta66, a specific inhibitor of Orai1 [26,27], on the Ca2+ mobilization produced by UTP in SKOV3 cells in N-Ca^2+^ conditions. Stimulation of SKOV-3 cells with a 10 μM UTP in the presence of a 500 nM Synta66 produced a transient increment in [Ca^2+^]_i_ but without the sustained component described previously (Figure 3E). Furthermore, these modifications of the Ca^2+^ kinetics were apparent when comparing the slope of the sustained component of the response (Figure 3F). These results demonstrate that the Ca^2+^ influx elicited by UTP in SKOV-e cells occurs due to Orai1 activity. 

### 2.5. Ca^2+^ Influx Due to SOCE Activation by Thapsigargin Is Inhibited by 2-APB in Metastatic SKOV-3 Cells

**Figure 4:**
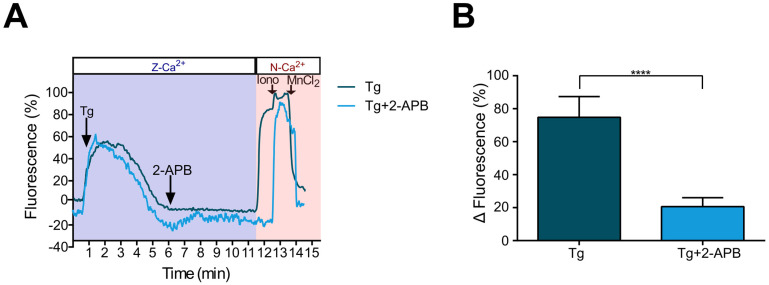
2-APB inhibits Ca^2+^ influx induced by calcium store depletion with thapsigargin in metastatic SKOV-3 cells. Ca^2+^ recording was performed in cultures of SKOV-3 cells loaded with Fluo-4AM. Thapsigargin (Tg) of 1 μM was added in the Z-Ca^2+^ extracellular solution; at minute 6 of the recording, 2-APB 50 μM was added, and after 5 min, extracellular Ca^2+^ was restored by the addition of the N-Ca^2+^ solution; at the end of the protocol, ionomycin (10 μM) and MnCl_2_ (5 mM) were sequentially applied to determine the maximum and minimum levels of intracellular Ca^2+^. (**A**) Representative traces of Ca^2+^ responses recorded under the described protocol and in control cultures without 2-APB addition. The arrows indicate the time of drug application. (**B**) Mean ± S.D. of the increase in [Ca^2+^]_i_ after extracellular Ca^2+^ restoring in the presence or absence of 2-APB. At least 100 cells were analyzed per experiment, n = 3. **** *p* < 0.0001, Student’s *t*-test.

So far, our data suggest that SOCE current is mainly present in metastatic SKOV-3 cells and that the absence of SOCE in CAOV-3 cells could be related to the differential expression of the ORAI1 protein. To confirm the presence of SOCE current in metastatic SKOV-3 cells, we employed 2-Aminoethyl Diphenyl Borate (2-APB), a widely known inhibitor of SOCE [28]. For these experiments, to elicit SOCE activation, intracellular Ca^2+^ stores were depleted by adding thapsigargin of 1 μM to the Z-Ca^2+^ extracellular solution; then, 50 μM of 2-APB was applied, and after 6 min, N-Ca^2+^ was restored; finally, maximum and minimum levels of [Ca^2+^]_i_ were estimated by addition of ionomycin (10 μM) and MnCl_2_ (25 mM), respectively (Figure 4A). N-Ca^2+^ restoring induced a significant increment in [Ca^2+^]_i_ (74.81 ± 0.94 ΔF) that was strongly decreased by the application of 2-APB (20.60 ± 0.85 ΔF) showing an inhibition of 72% (Figure 4A,B), confirming that Ca^2+^ influx observed during P2Y2 receptor activation by UTP depends on SOCE activation. 

### 2.6. UTP-Induced Ca^2+^ Influx Is Inhibited by 2-APB in Metastatic SKOV-3 Cells, Indicating SOCE Participation

**Figure 5:**
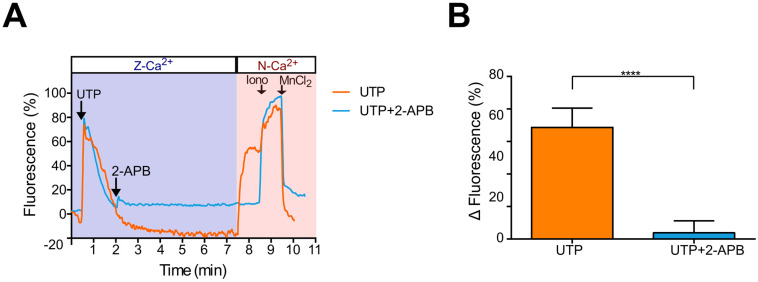
Binding of UTP to P2Y2 receptor elicits Ca^2+^ influx produced by SOCE activation. Ca^2+^ recording was performed in metastatic SKOV-3 cells loaded with Fluo-4 AM. Ca^2+^ storages were emptied by application of UTP of 10 μM in the Z-Ca^2+^ extracellular solution; at minute 2 of the recording, 2-APB of 50 μM was applied and then extracellular Ca^2+^ was restored by the addition of the N-Ca^2+^ solution to view the Ca^2+^ influx. (**A**) Representative traces of responses elicited under the described protocol and in control cells without 2-APB addition. (**B**) Mean ± S.D. of the [Ca^2+^]_i_ response magnitude detected after Ca^2+^ was restored. The arrows indicate the time of drug application. At least 100 cells were analyzed per experiment, n = 3. **** *p* < 0.0001, Student’s *t* test.

One of the aims of this work was to evaluate whether the SOCE current is an effector of P2Y2 receptor in OvCa. To test whether the P2Y2 receptor was a specific inductor of SOCE in SKOV-3 cells, cultures were stimulated in Z-Ca^2+^ with UTP of 10 μM; then, 2-APB of 50 μM was added, and after 5 min, N-Ca^2+^ was restored to show the Ca^2+^ influx as a result of SOCE activation. *n* the end, ionomycin and MnCl_2_ were added to determine maximum and minimum [Ca^2+^]_i_. In Z-Ca^2+^, UTP induced a fast transient (73.8 ± 0.9 ΔF) that reached the basal level after around 2 min; after 5 min of 2-APB addition, extracellular Ca^2+^ restoring to N-Ca^2+^ elicited a significant increment in [Ca^2+^]_i_ that was prevented by the presence of 2-APB (Figure 5). This observation confirms that SOCE can be elicited by P2Y2 activation with UTP.

### 2.7. Blocking of SOCE with 2-APB Inhibits UTP-Induced Cell Migration

SOCE-mediated Ca^2+^ entry is a key regulator of cell migration in breast cancer cells [20]. In OvCa cells, our group demonstrated that stimulation of SKOV-3 with UTP of 10 μM induced an increase in cell migration, an effect mediated by the activity of the P2Y2 receptor [16]; moreover, in the present work, we showed that UTP can activate SOCE. Therefore, we investigated whether P2Y2 receptor-dependent SOCE activation in OvCa-derived cells could have an effect on cell migration; to achieve this, we performed a wound-healing assay.

**Figure 6:**
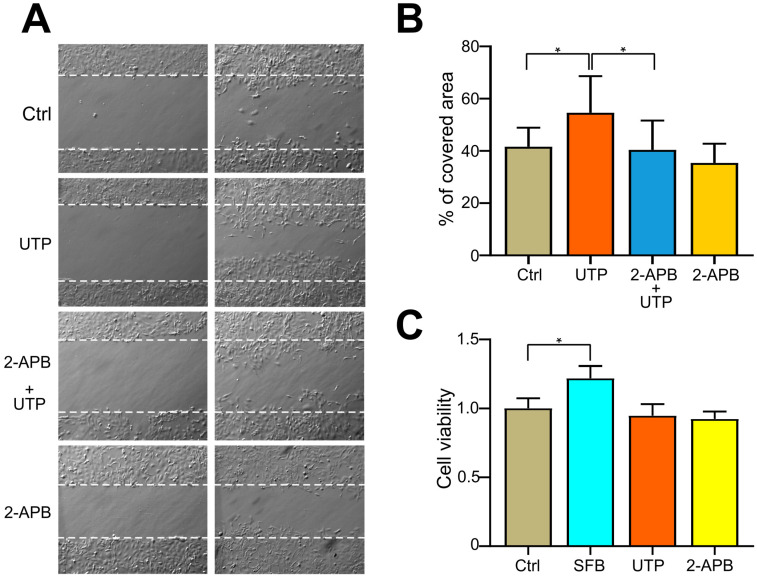
Inhibition of SOCE with 2-APB block UTP-induced cell migration. (**A**) Representative pictures from the wound healing assay, performed to evaluate the effect of a 1 μM 2-APB on 10 μM UTP-induced cell migration. (**B**) Mean ± S.D. of three independent experiments with five determinations each. (**C**) Cell viability was assessed by an MTS assay. * *p* < 0.05, ANOVA.

We analyzed the effect of pharmacological blocking of SOCE with 2-APB on UTP-induced cell migration in SKOV-3 cells. As expected, UTP (10 μM) induced an increment in cell migration (54.6 ± 3.7 vs. 41.6 ± 1.9% of covered area for UTP and control, respectively). Preincubation with a 1 μM 2-APB abolished the effect of UTP on cell migration (40.4 ± 2.8% of covered area) (Figure 6A,B). Similar results were obtained when carrying out the migration assay in Boyden chambers with 8 μm pores; UTP induced an increment in cell migration (217 ± 27% of number of cells/field of control) that was abolished by 2-APB (Figure S2). To rule out the contribution of cell duplication in the induction of cell migration by UTP, an MTS assay was also performed. Thus, a 10% fetal bovine serum (FBS), the positive control of this experiment, induced an increment in cell viability (1.21 ± 0.03-fold of control at 24 h), while a 10 μM UTP or 2-APB did not induce changes in cell viability (94.84 ± 0.03- and 92.38 ± 2.23-fold of control at 24 h, respectively) (Figure 6C). Importantly, UTP was unable to induce cell migration in non-metastatic line CAOV-3 (Figure S3).

## 3. Discussion

It is well established that elements of the purinergic system are expressed in a wide variety of cancers in many tissues and organs [6]. Furthermore, in recent years, it has been established that extracellular ATP is an important element of the TME that contributes to the tumor progression by receptor-dependent mechanisms (for in-depth reviews: [3,29,30,31]).

Ample evidence supports a key role of the P2Y2 receptor in tumor growth and its ability to spread and metastasize, as has been shown for lung [8], breast [14,15,32,33,34], prostate [11,12], ovary [9,16], liver [35,36], stomach [37], and pancreatic [38] cancers. Although some mechanisms mediating the actions of the P2Y2 receptor have been outlined, they are not fully understood, especially those involving Ca^2+^ mobilization. The aim of the present work was to demonstrate that P2Y2 receptor signaling induces SOCE in metastatic cell lines, with a possible role in the induction of cell migration. 

Although the relationship between the P2Y receptor and SOCE in cancer context has been poorly described, it was reported that P2Y6 receptor-dependent SOCE activity suppresses gastric cancer cell growth through a novel SOCE/Ca^2+^/β-catenin pathway [39]. However, in OvCa, this mechanism remains unexplored. Expression of UTP-sensitive P2Y2/P2Y4 receptors has already been demonstrated in OvCa cells (SKOV-3 and CAOV-3) [16] (Figure S1). Here, we show that UTP stimulation induces potent Ca^2+^ responses in both SKOV-3 (metastatic) and CAOV-3 (non-metastatic) OvCa cells. The main difference observed between the two lines was the presence of the UTP-dependent Ca^2+^ influx, which was detected only in SKOV-3 cells. This observation has important implications because the UTP-dependent Ca^2+^ influx in SKOV-3 cells generates an enhanced and sustained Ca^2+^ tone that could have physiological implications, since it is well described that calcium signaling is a critical regulator of cancer progression such as cell proliferation and invasiveness [40,41].

Based on the observations described, we investigated whether the Ca^2+^ influx in SKOV-3 cells could be mediated by SOCE activation. Thus, by intracellular Ca^2+^ recording techniques, we demonstrated that SOCE can be elicited in metastatic SKOV-3 cells by thapsigargin-mediated treatment in Z-Ca^2+^ extracellular conditions and become evident when restoring to normal extracellular Ca^2+^ (Figure 2). Furthermore, SOCE activation was abolished by 2-APB application (Figure 4 and Figure 5). This evidence shows that SOCE can be activated in SKOV-3 cells, and also that this metastatic cell line is more efficient than non-metastatic CAOV-3 in eliciting SOCE. 

Interestingly, it was evident that mRNA from healthy tissues expressed lower levels of transcripts coding for SOCE machinery (Figure 3A). The analysis of the expression of the SOCE machinery transcripts (Figure 3) suggested that this difference could be explained by the expression level of ORAI1, which is far more abundant in SKOV-3 cells than in CAOV-3 (Figure 3B–D). Furthermore, the use of Synta66, a specific inhibitor of ORAI1, suggested that this channel is essential for P2Y2-mediated activation of SOCE in metastatic ovarian carcinoma cells (Figure 3E,F). However, establishing whether the presence of SOCE and a low expression level of ORAI1 is a tissue signature of OvCa should be further addressed in future studies.

An important goal of this study was to determine the type of receptor mainly activated by UTP, and also test whether its activation could promote SOCE in OvCa cell lines. Our results showed that UTP responses in SKOV-3 and CAOV-3 were mainly mediated by the P2Y2 receptor, because the presence of ARC118925XX abrogated Ca^2+^ mobilization elicited by the nucleotide (Figure 1); indeed, P2Y2 receptors can activate SOCE since the Ca^2+^ influx (sustained phase of the response elicited by UTP) could be blocked by 2-APB (Figure S4). Interestingly, 10 μM of UTP is more potent than 1 µM of thapsigargin in inducing the depletion of intracellular Ca^2+^ stores (Figure S5), suggesting that purinergic agonists can act as efficient effectors in metastatic OvCa cells by inducing SOCE.

Previously, we demonstrated that P2Y2 receptor activation regulates cell migration in metastatic SKOV-3 cells [16]. Now, we generated results indicating that SOCE activation is an important mechanism that contributes to accomplishing this process. These data were supported when the UTP-dependent increase in cell migration was blocked by pharmacological SOCE inhibition (Figure 6 and Figure S2). Interestingly, UTP was unable to induce cell migration in non-metastatic CAOV-3 cells (Figure S3). Moreover, our observations agree with those of previous reports in other cancer systems [20,42], suggesting that SOCE activation plays essential roles in cancerous cells and, in consequence, represents a potential anticancer target.

To our knowledge, this is the first report describing SOCE as a P2Y2 receptor effector in the context of OvCa. The observation that both cell lines, non-metastatic (CAOV-3) and metastatic (SKOV-3), express functional P2Y2 receptor, but only SKOV-3 cells show a SOCE activated by this purinergic receptor, strongly suggest that ovarian oncogenic features could be related to the presence in the endoplasmic reticulum/plasma membrane of specialized SOCE-involved molecules. In this context, it is plausible to propose that increasing the concentration of ATP in an autocrine-paracrine manner in the TME might activate P2Y2 receptor and, consequently, SOCE to promote cell migration in metastatic ovarian carcinoma cells. The observations reported here reinforce the possibility that SOCE machinery could be a target for anticancer therapies.

## 4. Materials and Methods

### 4.1. Ovarian Carcinoma Cell Lines

CAOV-3 and SKOV-3 cells were purchased from the American Type Culture Collection (ATCC, Manassas, VI, USA). CAOV-3 cells were cultured in Dulbecco’s Modified Eagle Medium (DMEM) and SKOV-3 were cultured in Roswell Park Memorial Institute (RPMI) 1640 medium. Both were complemented with a 10% fetal bovine serum and a 1X antibiotic–antimycotic (100 U of penicillin, 100 μg of streptomycin and 0.25 ug of Fungizone per mL) (Thermo Scientific, Waltham, MA, USA). Cultures were maintained at 37 °C in an atmosphere of 5% CO_2_. For both lines, no more than 15 subculture passes were made.

### 4.2. Measurements of Intracellular Ca^2+^ in Single Cells

Cultured SKOV-3 or CAOV-3 cells were seeded onto coverslips and incubated for 30 min at 37 °C with 2 μM of Fluo-4 AM (Thermo Scientific, Waltham, MA, USA) in a Krebs solution (in mM: 150 NaCl, 1 KCl, 1.5 CaCl_2_, 1 MgCl_2_, 10 HEPES, and 4 glucose with 5% CO_2_ and 95% O_2_, pH adjusted to 7.4). After incubation, cells were washed to remove the unincorporated dye. Coverslips were mounted in a recording chamber standing on the platform of a Nikon eclipse Ts2R-FL inverted fluorescence microscope. Videos were acquired with a 20X microscope objective coupled to a Retiga Electro CCD camera with Ocular scientific image acquisition software (Teledyne Photometrics, Tucson, AR, USA) and images were collected every 500 ms. All the stimuli were applied manually with a pipette. When employed, inhibitors were pre-incubated between 5 and 15 min (depending on the mechanism of action of the drug) before the subsequent stimulus.

The recordings were performed in N-Ca^2+^: [Ca^2+^] = 1.5 mM or Z-Ca^2+^ (where CaCl_2_ was omitted and 2 mM EGTA was added): [Ca^2+^] = 2.4 × 10−11 M; Maxchelator (Webmaxc standard; UC, Davis).

Raw fluorescence intensity values were analyzed with Image J (National Institutes of Health, USA) and normalized using equation ((F/F0)-1)(100))/(Fmax), where F = fluorescence intensity measured at any given time, F0 = minimum fluorescence intensity obtained before the addition of any stimulus and Fmax = maximum fluorescence intensity obtained after adding 10 µM of ionomycin; these values were plotted vs. time.

### 4.3. Wound Closure Assay

Cell migration was evaluated by wound closure assay. SKOV-3 cells were cultured in 35 mm Petri dishes. When the monolayer reached confluence, a wound was made through the culture using a yellow pipette tip. The culture was then washed once to remove debris and the indicated pharmacological treatment was applied. Images were acquired with a 10X objective as the treatment was applied (t = 0) and after 16 h (t = 16, a time lesser than the duplication time for this cell line). Images were analyzed in ImageJ software (National Institutes of Health, Bethesda, MD, USA). Cell migration for each treatment was estimated by calculating the percentage of the initial wound area covered after 16 h. The experiments were performed four times. Five different areas were analyzed on each plate.

### 4.4. Cell Migration Assay in Boyden’s Chambers

For estimation of cell migration with Boyden’s chambers, cultures of SKOV-3 cells at 75–85% of confluence were fasted (8 h), then 5 × 10^4^ cells were placed in the upper compartment of Boyden´s chamber insert (Millicell-Millipore, Sigma-Aldrich, St. Louis, MO, USA) with the indicated pharmacological treatments diluted in serum and antibiotic free-RPMI medium. In the lower compartment, RPMI complemented with a 10% fetal bovine serum was placed as a chemoattractant. Chambers were incubated for 16 h. After this, the inserts were washed with a phosphate buffer (PBS, in mM: 136 NaCl, 2.7 KCl, 10 Na_2_HPO_4_, 1.8 KH_2_PO_4_, pH 7.4), fixed for 20 min in a 4% paraformaldehyde diluted in PBS and stained with a 0.5% crystal violet. Pictures were acquired with a Nikon eclipse Ts2R-FL inverted microscope. A total of 6 pictures of 3 independent experiments were counted using the ImageJ plugin Cell Counter (NCBI-USA).

### 4.5. Reverse Transcription, End-Point and Quantitative Polymerase Chain Reaction

Expression levels of STIM1, STIM2, ORAI1, ORAI2, ORAI3 and TRPC1 transcripts, all of them proteins supporting SOCE, were amplified by end-point PCR. Total RNA from hOSE cells was acquired from ScienceCell (Carlsbad, CA, USA) catalog #7315, while RNA from SKOV-3 and CAOV-3 was isolated using Trizol reagent (Thermo Scientific) following the manufacturer’s instructions. RNA concentration was measured in a Nanodrop 1000 spectrophotometer (Wilmington, DE, USA) and its integrity was assessed by gel electrophoresis. Reverse transcription reaction was performed with 200 U of Moloney Murine Leukemia Virus (M-MLV) reverse transcriptase (Promega, Madison, WI, USA) using 1 ug of total RNA treated with RNAase-free DNAse as template and 0.25 μg of oligo dT as a primer. End-point PCR reactions were made in a C100 Thermal Cycle (Biorad). Temperature cycles consisted of an initial hold of 60 s at 95 °C, and 30 cycles of 30 s at 95 °C, 30 s at the specific Tm and 30 s at 72 °C and a final hold at 12 °C. Oligonucleotide sequences, Tm and amplicon size used for PCR reactions are shown in Table 1.

Quantitative PCR analysis for the Orai1 transcript was performed using a SYBR green-containing master mix (LightCycler 480 SYBR Green I Master, Roche, Mannheim, Germany), 3 μL of 1:5 dilution of the cDNA and 0.5 μM of oligonucleotides, in a LightCycler 4.8 thermal cycler (Roche, Mannheim, Germany). Amplification protocol consisted of 600 s at 95 °C, 35 cycles of 10 s at 95 °C, 10 s at 60 °C and 12 s at 72 °C. Melting analysis of the reactions was performed with a 0.25 °C/s ramp from 55 to 95 °C.

### 4.6. Biotinylation of Plasma Membrane Proteins and Western Blot

To obtain a preparation enriched in plasma membrane proteins that enables the detection of ORAI1 by Western blot, cultures of CAOV-3 and SKOV-3 at 85% of confluence in 100 mm dishes were incubated with 300 μM of the not-permeable reactive sulfo-NHS-biotin EZ link (Thermo Scientific #A39256) diluted in PBS buffer for 20 min. After that, cells were lysed by addition of 500 μL of a TNTE 0.1% buffer (in mM: 150 NaCl, 0.5 EDTA, 50 Tris-pH7.4, 0.05% Tritón X-100 and protease inhibitors mix (Sigma-Aldrich)). The homogenates were centrifuged at 10,000 rpm for 10 min at 4 °C, the supernatants were recovered, and the protein concentration in this fraction was determined by the Lowry method. A volume of each supernatant containing 1 mg of total protein was incubated with 20 μL of a streptavidin–sepharose bead conjugated (#3419, Cell Signaling Technologies) for 90 min at room temperature. After that, the beads were washed 3 times with a PBS buffer, resuspended in 50 μL of a 2X Laemlli buffer (125 mM Tris-HCl pH 6.8, 140 mM sodium dodecyl sulfate, 0.03 mM bromphenol blue, 20% *v*/*v* glycerol and 2% *v*/*v* 2-mercaptoethanol) and boiled for 5 min.

Immunoblot was performed using previously published methods [16]. Briefly, 20 μL of the immunoprecipitated was fractioned by a 10% SDS-PAGE and transferred to a polyvinylidene fluoride (PVDF) membrane. Primary antibody against ORAI1 (SC-74778, Santa Cruz Biotechnology) was used at 1:250 dilution and incubated overnight, as a secondary antibody goat anti-rabbit conjugated to HRP (Thermo Scientific) was employed at a 1:5000 dilution. Positive immunoreactive proteins were detected by chemoluminiscence with the reactive Immobilon Western HRP Substrate Millipore (Sigma-Aldrich). Images were analyzed in ImageJ software (NIH-USA).

## Figures and Tables

**Table 1:** Sequence of oligonucleotides and amplicon size of PCR reactions.

Transcript	Oligonucleotides	Amplicon Size
P2Y2	Forward: CTG GTA GCG AGA ACA CTA AGG Reverse: GTC AAT ATC CTG AGC CCC TG	189 bp
STIM-1	Forward: CAC CAC AGC ACT TCC TAT TT Reverse: GGA AGT CAT GGC ATT GAG AG	216 bp
STIM-2	Forward: GAC ACT CTT CAG TGG TTG ATA Reverse: TAT GAG GTG GGC GTG TTA	220 bp
ORAI-1	Forward: CCT TCG GCC TGA TCT TTA TC Reverse: CCA AAG CAC TGG AAG GG	206 bp
ORAI-2	Forward: AAG GAG ATG GGA TGG AGA G Reverse: TAC CGA GTG GTG GTT AGA G	196 bp
ORAI-3	Forward: TGC CAC ACC CGA CTA AT Reverse: GAA ACA CCC AAA TCC CTC TAC	220 bp
TRPC-1	Forward: CTG TGG ATT ATT GGG ATG ATT TG Reverse: CAC CAG TGT AGG ATG GAA TG	207 bp
CYC-1	Forward: CTC CTG CCA CAG CAT GGA C Reverse: CAT GCC TAG CTC GCA CGA T	251 bp

## Data Availability

Data are available upon reasonable academic request.

## Supplementary Materials

The following supporting information can be downloaded at: https://www.mdpi.com/article/10.3390/ph16070944/s1, Figure S1. (**A**) PCR amplification of P2RY2 transcript with specific oligonucleotides from hOSE, SKOV-3 and CAOV-3 cDNAs. (**B**) Analysis of amplicon sequence obtained in A, in BLAST platform. (**C**) Relative abundance of *P2RY2* transcript in CAOV-3 and SKOV-3 cell lines evaluated by quantitative PCR. *** *p* < 0.001 Student´s *t*-test, N = 7; Figure S2. Inhibition of SOCE with 2-APB blocks UTP-induced cell migration of metastatic ovarian carcinoma SKOV-3 cells. In a Boyden’s chamber assay, SKOV-3 cells were treated with 10 μM UTP with or without preincubation with 1 μM 2-APB by 16h, as described in Methods. Graph shows the mean ± s.e.m. of the number of cells/field for each pharmacological treatment. Pictures show representative fields of each condition. Data were collected by counting 6 fields each from 3 independent experiments. * *p* > 0.05 Student´s-*t* test; Figure S3. Cell migration of non-metastatic CAOV-3 cells is not modified by UTP. CAOV-3 cells were treated with 10 μM UTP by 16 h and cell migration was evaluated by scratch assay as described in Methods. Graph shows the mean ± s.e.m. of the covered area. Pictures are representative images of each experimental condition. Data were obtained by 5 areas each from 3 independent experiments and were analyzed by Student´s-*t* test but not significative differences were observed; Figure S4. The Ca^2+^ influx produced by UTP in metastatic SKOV-3 cells is due to SOCE activation. Representative Ca^2+^ fluorescence (Fluo-4) traces from SKOV-3 cells obtained in N-Ca^2+^ plus 2-APB. Cells were stimulated with 10 µM UTP in N-Ca^2+^ extracellular solution, in presence or absence of 2-APB 50 µM; at the end of the protocol, ionomycin (10 µM) and MnCl2 (5 mM) were sequentially applied to determine the maximum and minimum levels of intracellular Ca^2+^, respectively. At least 100 cells were analyzed per experiment, n = 3; Figure S5. UTP is a potent inducer of intracellular Ca^2+^ reservoir depletion in SKOV-3 cells. Representative Ca^2+^ recordings performed in cultures of SKOV-3 cells loaded with Fluo-4 AM. Thapsigargin (Tg) 1 μM (A) or UTP 10 μM (B) was added in Z-Ca^2+^ extracellular solution at 30 s; then when the Ca^2+^ returned to basal levels, UTP 10 μM (A) or (Tg) 1 μM was added; at the end of the protocol extracellular Ca^2+^ was restored by addition of N-Ca^2+^ solution, and ionomycin (10 μM) and MnCl_2_ (5 mM) were sequentially applied to determine the maximum and minimum levels of intracellular Ca^2+^. At least 100 cells were analyzed per experiment, n = 3.
