# Supplementary material for: Purinergic Activation of Store-Operated Calcium Entry (SOCE) Regulates Cell Migration in Metastatic Ovarian Cancer Cells
Source: Pharmaceuticals (Basel). 2023 Jun 29;16(7):944. doi: 10.3390/ph16070944 (PMC10384695; doi:10.3390/ph16070944)
Supplement: Supplementary file 1 [file pharmaceuticals-16-00944-s001.zip › pharmaceuticals-2366651-supplementary.pdf]

Supplementary figures

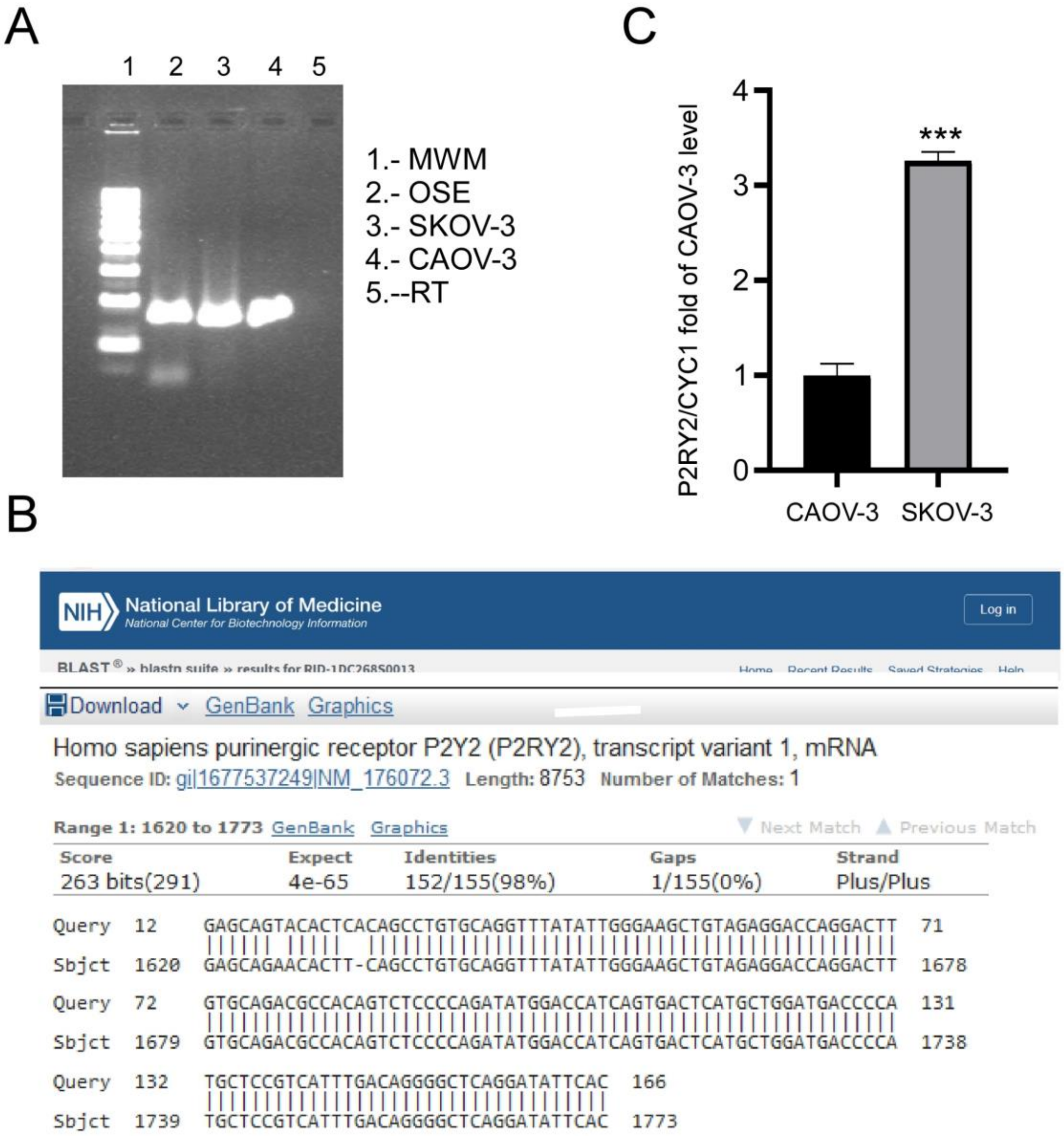

Figure S1. A) PCR amplification of P2RY2 transcript with specific oligonucleotides from hOSE, SKOV-3 and CAOV-3 cDNAs. B) Analysis of amplicon sequence obtained in A, in BLAST platform. C) Relative abundance of *P2RY2* transcript in CAOV-3 and SKOV-3 cell lines evaluated by quantitative PCR. ***p<0.001 Student´s t-test, N=7.

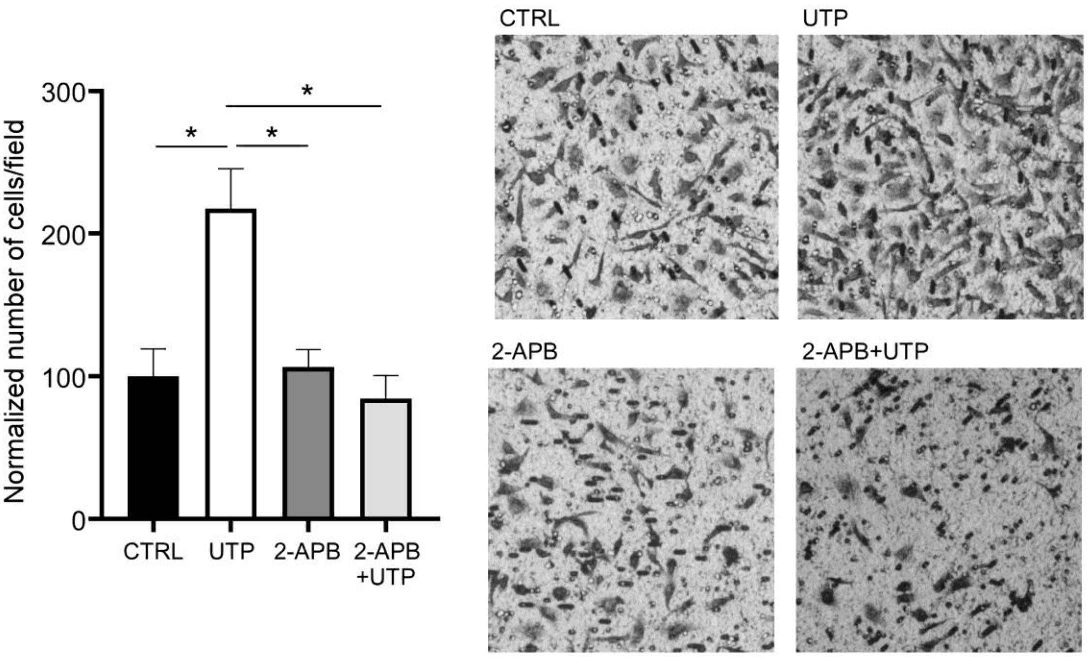

Figure S2. Inhibition of SOCE with 2-APB blocks UTP-induced cell migration of metastatic ovarian carcinoma SKOV-3 cells. In a Boyden´s chamber assay, SKOV-3 cells were treated with 10 μM UTP with or without preincubation with 1 μM 2-APB by 16h, as described in Methods. Graph shows the mean ± s.e.m. of the number of cells/field for each pharmacological treatment. Pictures show representative fields of each condition. Data were collected by counting 6 fields each from 3 independent experiments. *$p>0.05$ Student´s-t test.

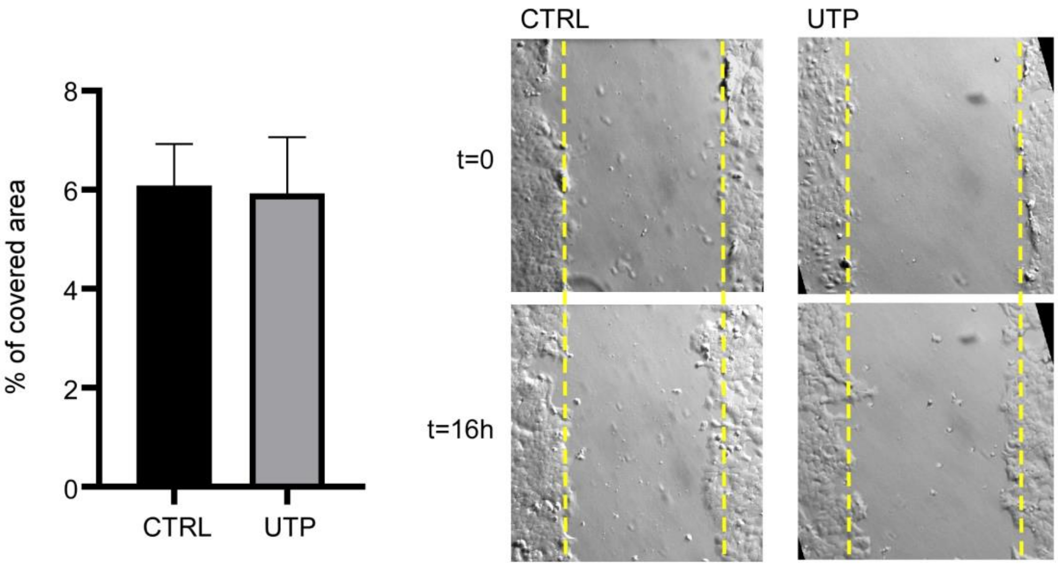

Figure S3. Cell migration of non-metastatic CAOV-3 cells is not modified by UTP. CAOV-3 cells were treated with 10 μM UTP by 16 h and cell migration was evaluated by scratch assay as described in Methods. Graph shows the mean ± s.e.m. of the covered area. Pictures are representative images of each experimental condition. Data were obtained by 5 areas each from 3 independent experiments and were analyzed by Student´s-t test but not significative differences were observed.

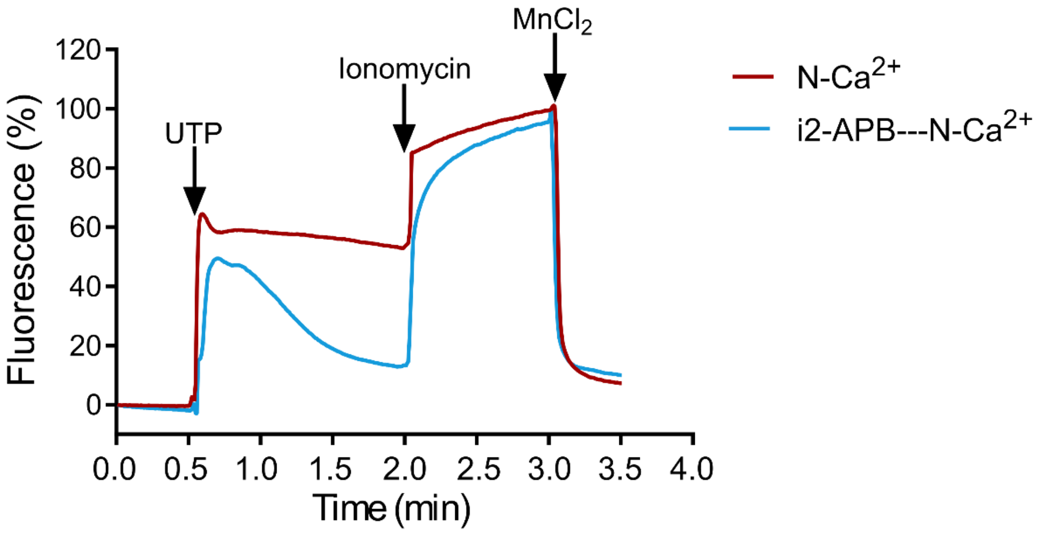

Figure S4. The Ca^{2+} influx produced by UTP in metastatic SKOV-3 cells is due to SOCE activation. Representative Ca^{2+} fluorescence (Fluo-4) traces from SKOV-3 cells obtained in N-Ca^{2+} plus 2-APB. Cells were stimulated with 10 µM UTP in N-Ca^{2+} extracellular solution, in presence or absence of 2-APB 50 µM; at the end of the protocol, ionomycin (10 µM) and MnCl2 (5 mM) were sequentially applied to determine the maximum and minimum levels of intracellular Ca^{2+}, respectively. At least 100 cells were analyzed per experiment, n=3.

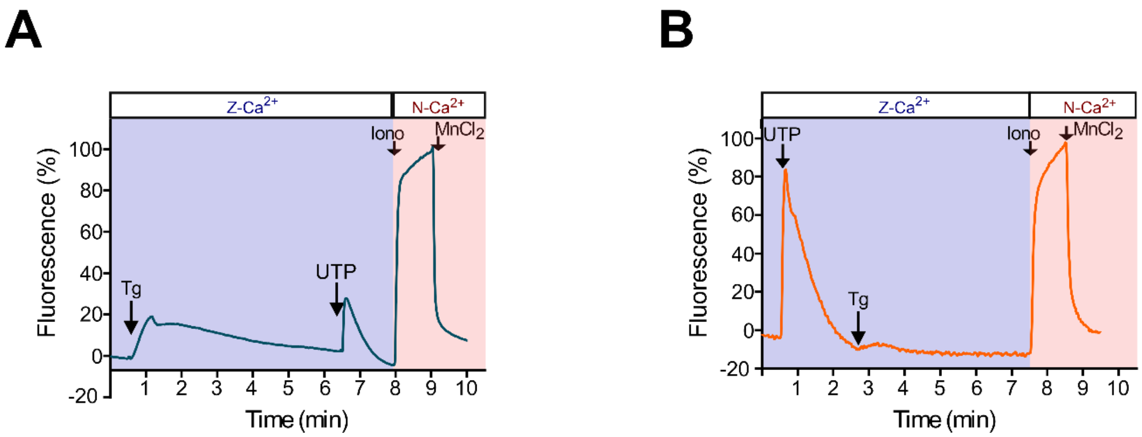

Figure S5. UTP is a potent inducer of intracellular Ca^{2+} reservoir depletion in SKOV-3 cells. Representative Ca^{2+} recordings performed in cultures of SKOV-3 cells loaded with Fluo-4 AM. Thapsigargin (Tg) 1 µM (A) or UTP 10 µM (B) was added in Z-Ca^{2+} extracellular solution at 30 s; then when the Ca^{2+} returned to basal levels, UTP 10 µM (A) or (Tg) 1 µM was added; at the end of the protocol extracellular Ca^{2+} was restored by addition of N-Ca^{2+} solution, and ionomycin (10 µM) and $MnCl_2$ (5 mM) were sequentially applied to determine the maximum and minimum levels of intracellular Ca^{2+}. At least 100 cells were analyzed per experiment, n=3.
